# Shoulder range of movement in the general population: age and gender stratified normative data using a community-based cohort

**DOI:** 10.1186/s12891-020-03665-9

**Published:** 2020-10-12

**Authors:** Tiffany K. Gill, E. Michael Shanahan, Graeme R. Tucker, Rachelle Buchbinder, Catherine L. Hill

**Affiliations:** 1 Adelaide Medical School, Faculty of Health and Medical Sciences, The University of Adelaide, Level 7, SAHMRI, North Tce, Adelaide, SA 5000 Australia; 2 Rheumatology Unit, Flinders Medical Centre, Southern Adelaide Local Health Network, Bedford Park, SA 5042 Australia; 3 College of Medicine and Public Health, Flinders University, Bedford Park, SA 5042 Australia; 4 Monash Department of Clinical Epidemiology, Cabrini Institute, Malvern, Vic 3144 Australia; 5 Department of Epidemiology and Preventive Medicine, School of Public Health & Preventive Medicine, Monash University, Melbourne, Vic 3004 Australia; 6 Rheumatology Unit, The Queen Elizabeth Hospital, Central Adelaide Local Health Network, Woodville, SA 5011 Australia

**Keywords:** Shoulder, Population, Range of motion

## Abstract

**Background:**

An understanding of the average range of movement of the shoulder that is normally achievable is an important part of treatment for shoulder disorders. The average range of active shoulder flexion, abduction and external rotation was measured in a population cohort aged 20 years and over without shoulder pain and/or stiffness in order to provide normative shoulder range data.

**Methods:**

Cross-sectional analysis using participants in a community-based longitudinal cohort study. There have been three stages of data collection – Stage 1 (1999–2003), Stage 2 (2004–2006) and Stage 3 (2008–2010). Each stage has consisted a of broad ranging computer assisted telephone interview, a self-complete questionnaire and a clinic assessment. Participants in this study are those who undertook assessments in Stage 2. The main outcome measures were active shoulder range of movement (flexion, abduction and external rotation) measured as part of the clinic assessment using a Plurimeter V inclinometer. Mean values were determined and analyses to examine differences between groups (sex and age) were undertaken using non-parametric tests.

**Results:**

There were 2404 participants (51.5% male), mean age 45.8 years (SD 17.3, range 20–91). The average range of active right shoulder flexion was 161.5° for males and 158.5° for females, and active right shoulder abduction was 151.5° and 149.7° for males and females respectively. Shoulder range of movement declined with age, with mean right active shoulder flexion decreasing by 43° in males and 40.6° in females and right active shoulder abduction by 39.5° and 36.9° respectively. External rotation range also declined, particularly among females.

**Conclusion:**

To our knowledge this is the largest community-based study providing normative data for active shoulder range of movement. This information can be used to set realistic goals for both clinical practice and clinical trials.

## Introduction

Shoulder problems are a common cause of disability in the community, are often chronic and have a significant impact on the ability to undertake activities [1–3]. Their assessment and management comprise a significant proportion of general practice encounters [4]. In 2014–15, shoulder complaints were the third most common musculoskeletal problem seen by general practitioners (GPs) in Australia, behind back and knee problems [4]. People with shoulder complaints also commonly visit physiotherapists, either as the first point of call or following referral from a GP [4].

An important part of the clinical assessment is to determine a person’s range of active movement, the degree to which a person can move their shoulder in different directions, as this will assist in making a diagnosis and provide valuable information about a person’s functional limitations [5]. There are generally considered to be six movements of the shoulder: flexion, extension, abduction, adduction, external and internal rotation. Each movement contributes in different ways to the ability to undertake activities of daily living. For example, a combination of adduction and flexion are required to reach up to a high shelf or wash hair. Internal rotation, extension and adduction are required to reach into the back pocket; and abduction, flexion and external rotation are required to comb hair.

Normal range of active movement of the shoulder has been specified by the American Academy of Orthopedic Surgeons (AAOS) to be 180° for flexion and abduction  and 90° for external rotation [6]. However the population and methods used to obtain these values, and potential measurement error have not been described [7, 8], and there have been no published studies of normative values in samples representative of the general population. Shoulder range of movement (ROM) may be influenced by age, sex, work history and hand dominance, in addition to the measurement equipment being used and the assessor variability [9–14]. In terms of chronic diseases that impact ROM, people with diabetes have a higher reported prevalence of shoulder pain with subsequently increased levels of disability and reductions in activities of daily living [15, 16], however one community-based study has also reported reduced average ranges of active flexion, abduction and external rotation, even among those with diabetes without a history of shoulder pain and/or stiffness [17]. Hill et al. have also compared the active flexion, abduction and external rotation range of those with and without shoulder pain and/or stiffness (adjusted for age, sex, body mass index and current smoking) and demonstrated that while those without pain or stiffness have a greater ROM, their average range of active flexion was less than 180° and external rotation less than 90° [18].

Due to the highlighted lack of data, the aim of this study was to determine normative data of active range of shoulder flexion, abduction and external rotation among a large population-based sample of community dwelling adults aged over 20 years without a history of shoulder pain or stiffness. Differences in range according to age, sex, hand dominance and presence of diabetes (adjusted for age and sex) were also investigated.

## Methods

### Study design

Participants were recruited from the North West Adelaide Study (NWAHS), a longitudinal cohort study of 4056 randomly selected adults aged 18 years and over at the time of recruitment.

### Study setting

The cohort was selected from the northern and western regions of Adelaide, South Australia. This sample region represents approximately half of the metropolitan area of Adelaide (total population of approximately 1.2 million) and almost one-third of the population in South Australia (population of approximately 1.6 million) [19]. The study commenced in 1999 to 2003 with Stage 1, Stage 2 was conducted between 2004 and 2006 and Stage 3 was conducted between 2008 and 2010, with the aim of providing longitudinal measured and self-reported data to assist in increasing the ability of strategies and policies to prevent, detect and manage a range of chronic conditions [20]. Data were collected for the study using a Computer Assisted Telephone Interview (CATI), a self-completed questionnaire and a clinic assessment at each stage [20, 21].

### Participants

Participants in this study were those who had already participated in Stage 1 of data collection between 1999 and 2003 and then were recontacted to be involved in the Stage 2 data collection in 2004 to 2006. Shoulder ROM was measured as part of the Stage 2 clinic data collection and thus all participants who attended this assessment had shoulder ROM assessed. However, as part of the CATI questionnaire which was also a component of the Stage 2 assessment, participants were asked if they had ever had shoulder pain or stiffness, or if they had ever been told by a doctor that they had rheumatoid arthritis. Participants who responded “yes” to one or more of these questions were excluded from the shoulder ROM analysis.

### Ethics approval

Ethics approval for the study was obtained from the Human Research Ethics Committee of The Queen Elizabeth Hospital, Adelaide, South Australia (Application number 2004030) and all participants provided written informed consent.

### Variables and measurements

Clinical staff with a medical/nursing background performed the clinic assessment including measurement of range of active flexion, abduction and external rotation of both shoulders. Due to time constraints in the clinic, only one measurement was recorded, however this also avoided potential fatigue from multiple measurements or stretching of the joint and a possible increase in range [22]. An experienced anthropometrist trained staff in a standardised protocol using a Plurimeter V inclinometer to measure active flexion (forward elevation) and abduction (side elevation) in standing to the nearest degree [22]. The inclinometer is gravity-referenced and thus calibrated on the basis of gravity. As a result, the placement error is minimized as the movement starting position is fixed [22]. Green et al. [22] also demonstrated that the intra- and inter-rater intraclass correlation coefficients (ICCs) were excellent for shoulder flexion and abduction using the Plurimeter V inclinometer. External rotation, also measured in standing, was assessed visually to the nearest five degrees as this has previously been shown to be comparable to goniometric measurements [23, 24]. The participant commenced with the upper arm by their side, bent at the elbows and then the arm was externally rotated.

Other variables used as part of the analysis were age, sex, presence of diabetes and hand dominance. Participant age was determined from date of birth to the date of the clinic attendance and categorised into five-year age groups. Diabetes was defined by self-reported doctor- diagnosed diabetes and/or a fasting plasma glucose level of ≥7.0 mmol/l performed at the clinic visit and hand dominance was also recorded (participants were asked “What is your dominant hand?”).

### Data weighting

In Stage 1, data were weighted by region (western and northern health regions of Adelaide), age group, sex and probability of selection in the household to the Australian Bureau of Statistics 1999 Estimated Resident Population and the 2001 Census data. Stage 2 was reweighted using the 2004 Estimated Resident Population for South Australia, incorporating participation in the three components, while retaining the original weight from Stage 1 in the calculation. All analyses in this paper, where applicable, are weighted to the population of the northern and western suburbs of Adelaide.

### Statistical analyses

Statistical analyses were conducted using IBM Statistics SPSS version 24 (IBM, Armonk, NY, USA). Mean and median values for sex and each age group were determined. The overall mean and median values according to hand dominance was also calculated. Analysis was undertaken using the Kolmogorov-Smirnov test to assess whether the data were normally distributed. Data were not normally distributed and as a result, differences between sex, age groups and hand dominance were examined using the Mann-Whitney U and Kruskal Wallis tests as appropriate. Differences between the right and left sides ROM were examined using the Wilcoxon signed rank test. The significance level of tests (alpha) was set at 5%. The mean range of active movement for those with and without diabetes was examined using linear regression and multiple analysis of variance (MANOVA), in order to provide a marginal mean adjusted by age and sex.

## Results

A total of 3175 participants provided active shoulder flexion, abduction and external rotation measurements. Overall, 771 were excluded from the analysis as they had reported a history of having had shoulder pain or stiffness and/or a history of doctor-diagnosed rheumatoid arthritis. Of the remaining 2404 participants, 1237 (51.5%) were male. The mean age was 45.8 years (SD 17.3, range 20–91). Hand dominance was recorded for 2382 participants and 10.8% (*n* = 257) were left-handed. Diabetes status was available for 2379 participants and its prevalence was 6.3% (*n* = 150).

### Shoulder flexion

Table 1 shows the mean and range of active shoulder flexion by sex and shoulder. Overall, the mean range for males was 159.9° (SD 18.1) for the left side and 161.5° (SD 18.7) for the right side. These values were slightly greater than those obtained for females (157.1° [SD 18.2] and 158.5° [SD 19.1] for the left and right sides respectively). Active flexion declined with age in both sexes. Overall, there was a statistically significant difference in the range of active shoulder flexion between the right and left shoulders, with the right shoulder having greater range (*p* < 0.001). For the right shoulder, there was a statistically significant difference in active range of flexion between males and females, and across age groups (p < 0.001). There was a similar pattern for the left shoulder (p < 0.001). When those with diabetes were excluded from the analysis, overall mean range of left and right active flexion for males was 160.7° (SD 17.8°) and 162.2° (SD 18.5°), and for females 158.0° (SD 17.7°) and 159.3° (SD 18.6°) respectively.

**Table 1:** Mean (SD), median (IQR) and range of active shoulder flexion in degrees for males and females by five-year age groups

		Left shoulder			Right shoulder		
		Mean (SD)	Median (IQR)	Range	Mean (SD)	Median (IQR)	Range
**All participants**	**Male (*n*** **= 1237)**	159.9 (18.1)	162.0 (152–174)	0–180	161.5 (18.7)	165.0 (155–175)	14–180
	**Female (*n*** **= 1167)**	157.1 (18.2)	160.0 (150–170)	20–180	158.5 (19.1)	160.0 (150–171)	0–180
**20–24 years**	**Male (*n*** **= 97)**	168.6 (13.1)	174.0 (160–178)	130–180	170.8 (18.2)	179.0 (170–180)	90–180
	**Female (*n*** **= 99)**	166.4 (10.1)	169.0 (160–174)	146–180	164.7 (11.1)	165.8 (160–174)	140–180
**25–29 years**	**Male (*n*** **= 170)**	165.0 (10.1)	162.0 (158–175)	142–180	165.2 (9.8)	164.0 (160–174)	145–180
	**Female (*n*** **= 119)**	164.8 (12.8)	166.0 (152–180)	138–180	166.0 (13.1)	170.0 (155–180)	136–180
**30–34 years**	**Male (*n*** **= 146)**	166.2 (12.9)	170.0 (160–176)	130–180	167.9 (11.5)	170.0 (160–177)	123–180
	**Female (*n*** **= 145)**	162.9 (12.2)	162.0 (156–172)	130–180	164.4 (13.1)	166.3 (156–173)	100–180
**35–39 years**	**Male (*n*** **= 131)**	162.3 (21.7)	166.0 (160–176)	40–180	162.8 (24.3)	168.0 (160–176)	20–180
	**Female (*n*** **= 122)**	165.2 (13.3)	168.0 (158–180)	133–180	166.2 (12.5)	168.0 (158–180)	132–180
**40–44 years**	**Male (*n*** **= 140)**	160.9 (14.2)	160.0 (152–170)	100–180	165.5 (13.1)	166.0 (156–174)	120–180
	**Female (*n*** **= 125)**	160.2 (13.7)	160.0 (150–170)	118–180	163.7 (14.0)	164.0 (156–176)	114–180
**45–49 years**	**Male (*n*** **= 113)**	162.9 (12.4)	162.0 (155–174)	110–180	164.9 (13.1)	168.0 (160–176)	120–180
	**Female (*n*** **= 105)**	158.0 (13.3)	160.0 (150–166)	124–180	159.9 (14.0)	160.0 (150–170)	110–180
**50–54 years**	**Male (*n*** **= 94)**	163.6 (16.7)	167.1 (154–178)	110–180	165.0 (18.6)	170.0 (160–176)	50–180
	**Female (*n*** **= 89)**	158.0 (14.5)	160.0 (150–168)	116–180	160.1 (14.5)	160.0 (150–170)	122–180
**55–59 years**	**Male (*n*** **= 86)**	157.3 (15.1)	160.0 (149–170)	120–180	159.4 (15.6)	160.0 (150–171)	100–180
	**Female (n = 86)**	154.7 (15.5)	154.0 (144–168)	110–180	157.1 (14.6)	160.0 (149–170)	120–180
**60–64 years**	**Male (*n*** **= 58)**	155.9 (15.2)	159.5 (144–169)	110–180	157.4 (16.5)	160.0 (150–170)	115–180
	**Female (*n*** **= 55)**	146.0 (26.1)	150.0 (134–160)	20–180	146.5 (27.1)	150.0 (140–163)	10–180
**65–69 years**	**Male (*n*** **= 62)**	149.9 (20.1)	151.8 (140–162)	60–180	152.3 (20.1)	156.1 (144–162)	78–180
	**Female (*n*** **= 61)**	151.6 (18.0)	153.9 (143–160)	78–180	152.1 (15.7)	152.0 (144–162)	80–180
**70–74 years**	**Male (*n*** **= 49)**	143.3 (27.2)	150.0 (130–160)	0–180	146.8 (19.3)	147.5 (130–161)	100–180
	**Female (*n*** **= 54)**	145.9 (17.9)	150.0 (136–160)	40–180	144.8 (29.7)	150.8 (131–162)	0–180
**75–79 years**	**Male (*n*** **= 52)**	143.0 (18.7)	142.0 (133–158)	50–174	143.4 (26.1)	145.1 (130–160)	14–178
	**Female (*n*** **= 46)**	138.1 (21.0)	141.8 (130–153)	52–175	141.9 (21.5)	145.0 (136–152)	60–172
**80–84 years**	**Male (*n*** **= 31)**	137.1 (24.1)	140.0 (123–151)	50–174	140.2 (22.0)	142.4 (125–156)	72–180
	**Female (*n*** **= 51)**	132.1 (25.2)	132.0 (119–149)	64–180	133.7 (24.3)	140.0 (120–150)	66–178
**85 years and over**	**Male (*n*** **= 10)**	129.6 (23.2)	136.1 (112–150)	90–158	127.8 (24.4)	123.7 (111–151)	90–174
	**Female (*n*** **= 10)**	129.9 (30.9)	138.0 (100–154)	80–170	124.1 (39.6)	130.0 (80–160)	70–174

### Shoulder abduction

Table 2 shows the mean and range of active abduction by sex and shoulder. Overall, the mean range for males was 149.7° (SD 20.3) for the left side and 151.5° (SD 20.1) for the right side and for females 147.7° (SD 20.0) and 149.7° (SD 20.3) for the left and right sides respectively. Active abduction was slightly greater for males compared to females and higher in the right shoulder compared to the left. Active abduction declined with age in both sexes. Overall, there was a statistically significant difference in the range of active shoulder abduction between the right and left shoulders (*p* < 0.001). There was a statistically significant difference in active range of abduction between males and females, and across age groups for the right shoulder (p < 0.001). There was a similar pattern for the left shoulder (p < 0.001). When those with diabetes were excluded from the analysis the overall mean active abduction range in the left and right shoulder in males was 150.6° (SD 19.6°) and 152.4° (SD 19.2°), and for females 148.6° (SD 19.4°) and 150.6° (SD 19.8°) respectively.

**Table 2:** Mean (SD), median (IQR) and range of active shoulder abduction in degrees for males and females by five-year age groups

			Left shoulder		Right shoulder		
		Mean (SD)	Median (IQR)	Range	Mean (SD)	Median (IQR)	Range
**All participants**	**Male (*n*** **= 1237)**	149.7 (20.3)	152.0 (140–162)	0–180	151.5 (20.1)	156.0 (142–164)	0–180
	**Female (*n*** **= 1167)**	147.7 (20.0)	150.0 (140–160)	22–180	149.7 (20.3)	152.0 (140–162)	0–180
**20–24 years**	**Male (*n*** **= 97)**	158.8 (21.7)	162.3 (154–172)	90–180	158.4 (25.0)	164.0 (160–172)	78–180
	**Female (*n*** **= 99)**	156.0 (13.8)	158.0 (140–168)	126–174	156.4 (12.1)	160.0 (150–166)	128–170
**25–29 years**	**Male (*n*** **= 170)**	153.4 (14.0)	153.4 (142–164)	122–180	154.1 (13.6)	160.0 (143–164)	124–180
	**Female (*n*** **= 119)**	155.2 (13.3)	150.0 (146–164)	124–180	157.3 (15.3)	158.4 (150–169)	120–180
**30–34 years**	**Male (*n*** **= 146)**	156.1 (14.2)	160.0 (150–164)	100–178	157.0 (15.7)	160.0 (152–166)	90–180
	**Female (*n*** **= 145)**	155.9 (12.9)	158.0 (150–160)	110–180	156.0 (12.7)	156.0 (150–165)	100–180
**35–39 years**	**Male (*n*** **= 131)**	153.4 (20.7)	158.0 (148–165)	50–180	155.1 (21.3)	160.0 (150–166)	40–180
	**Female (*n*** **= 122)**	156.4 (13.0)	158.0 (150–166)	100–180	158.8 (12.3)	160.0 (150–168)	120–180
**40–44 years**	**Male (*n*** **= 140)**	151.6 (16.2)	154.0 (144–160)	82–180	154.9 (14.3)	158.0 (146–166)	100–180
	**Female (*n*** **= 125)**	152.5 (15.1)	154.0 (142–163)	98–180	154.9 (15.4)	158.0 (148–164)	66–180
**45–49 years**	**Male (*n*** **= 113)**	152.4 (14.1)	154.0 (148–161)	78–180	154.5 (14.0)	156.0 (148–164)	104–180
	**Female (*n*** **= 105)**	148.5 (14.5)	150.0 (140–160)	90–180	151.1 (14.9)	152.0 (140–160)	100–180
**50–54 years**	**Male (*n*** **= 94)**	154.6 (16.2)	160.0 (150–164)	74–180	158.1 (15.9)	160.0 (154–166)	60–180
	**Female (*n*** **= 89)**	149.3 (15.0)	150.0 (142–158)	90–180	151.4 (16.9)	154.0 (141–161)	76–180
**55–59 years**	**Male (*n*** **= 86)**	146.5 (19.2)	150.0 (138–160)	60–179	148.6 (18.8)	150.0 (140–160)	60–180
	**Female (*n*** **= 86)**	146.2 (14.7)	148.0 (138–157)	100–180	149.6 (13.7)	150.0 (140–160)	110–176
**60–64 years**	**Male (*n*** **= 58)**	145.0 (18.9)	148.0 (140–154)	76–180	145.9 (18.1)	148.0 (139–159)	74–180
	**Female (*n*** **= 55)**	138.2 (23.5)	142.0 (130–154)	32–173	138.8 (26.5)	142.0 (130–155)	20–180
**65–69 years**	**Male (*n*** **= 62)**	135.7 (28.4)	140.0 (130–154)	0–170	137.5 (28.9)	142.0 (130–153)	0–180
	**Female (*n*** **= 61)**	140.5 (19.3)	142.0 (132–151)	70–176	142.6 (17.7)	144.0 (135–155)	72–175
**70–74 years**	**Male (*n*** **= 49)**	134.8 (27.4)	141.6 (130–152)	0–174	137.2 (21.7)	141.3 (127–153)	52–174
	**Female (*n*** **= 54)**	131.8 (22.9)	138.0 (120–150)	22–170	132.7 (30.9)	141.8 (120–152)	0–172
**75–79 years**	**Male (*n*** **= 52)**	134.2 (17.3)	138.0 (123–147)	70–164	136.4 (18.1)	140.0 (128–150)	70–178
	**Female (*n*** **= 46)**	127.8 (23.5)	130.0 (120–142)	32–170	133.3 (26.2)	140.0 (128–150)	36–165
**80–84 years**	**Male (*n*** **= 31)**	125.0 (27.1)	129.0 (118–144)	30–178	130.5 (22.8)	132.0 (120–150)	78–180
	**Female (*n*** **= 51)**	115.0 (28.7)	121.2 (90–135)	32–166	120.9 (26.5)	124.5 (100–140)	40–164
**85 years and over**	**Male (n = 10)**	119.7 (22.2)	121.7 (100–139)	80–160	118.9 (23.4)	111.9 (100–144)	80–160
	**Female (*n*** **= 10)**	118.9 (28.8)	112.0 (100–152)	80–160	119.5 (35.3)	120.0 (105–158)	60–160

### Shoulder external rotation

In contrast to active flexion and abduction, females had higher mean active external rotation compared to males, slightly greater in the right shoulder (Table 3). With age, active external rotation also declined but this was observed to be much greater for females. Again there was an overall statistically significant difference between the active range of the left and right sides (*p* < 0.001). In the right shoulder there was a statistically significant difference between males and females, and between age groups (p < 0.001). A similar pattern was evident for the left shoulder (p < 0.001). When those with diabetes were excluded from the analysis the overall mean range of left shoulder external rotation for males was 55.7° (SD 17.8°) and 57.5° (SD 17.4°) for right shoulder external rotation. For females, active left shoulder external rotation was 58.8° (SD 17.4°) and right was 61.2° (SD 17.7°) respectively.

**Table 3:** Mean (SD), median (IQR) and range of active shoulder external rotation in degrees for males and females by five-year age groups

		Left shoulder			Right shoulder		
		Mean (SD)	Median (IQR)	Range	Mean (SD)	Median (IQR)	Range
**All participants**	**Male (*n*** **= 1237)**	55.0 (17.9)	50.0 (45–70)	0–90	56.8 (17.6)	55.0 (45–70)	0–90
	**Female (*n*** **= 1167)**	58.5 (17.7)	60.0 (45–75)	0–90	60.9 (17.9)	60.0 (45–75)	0–90
**20–24 years**	**Male (*n*** **= 97)**	58.9 (18.4)	60.0 (45–75)	25–85	62.3 (15.9)	65.0 (47–75)	30–85
	**Female (*n*** **= 99)**	72.9 (13.2)	75.0 (65–80)	40–90	73.8 (14.7)	80.0 (70–85)	40–90
**25–29 years**	**Male (*n*** **= 170)**	62.4 (18.8)	60.0 (50–80)	25–90	64.5 (17.7)	65.0 (50–80)	30–90
	**Female (*n*** **= 119)**	67.3 (15.0)	70.0 (55–80)	35–90	72.5 (12.5)	75.0 (65–80)	40–90
**30–34 years**	**Male (*n*** **= 146)**	57.9 (16.6)	55.0 (45–75)	20–90	59.1 (16.5)	60.0 (45–75)	15–90
	**Female (*n*** **= 145)**	61.9 (14.3)	60.0 (50–75)	30–90	65.1 (13.8)	70.0 (55–75)	30–90
**35–39 years**	**Male (*n*** **= 131)**	56.2 (17.0)	55.0 (45–70)	20–90	56.3 (17.1)	55.0 (45–70)	10–90
	**Female (*n*** **= 122)**	60.5 (15.8)	60.0 (45–72)	30–90	62.5 (16.5)	65.0 (45–75)	30–90
**40–44 years**	**Male (*n*** **= 140)**	53.7 (15.8)	50.0 (45–65)	20–90	54.9 (15.7)	50.0 (45–67)	10–90
	**Female (*n*** **= 125)**	56.8 (16.5)	55.0 (45–70)	0–85	58.5 (17.7)	55.0 (45–75)	0–90
**45–49 years**	**Male (*n*** **= 113)**	53.0 (18.0)	50.0 (44–65)	10–90	53.8 (17.3)	50.0 (45–70)	20–85
	**Female (*n*** **= 105)**	54.5 (16.5)	50.0 (45–70)	20–90	56.1 (17.4)	50.0 (45–70)	15–90
**50–54 years**	**Male (*n*** **= 94)**	54.7 (17.2)	55.0 (40–70)	5–90	56.5 (17.8)	56.3 (45–73)	10–90
	**Female (*n*** **= 89)**	55.4 (16.4)	55.0 (45–70)	20–90	58.1 (16.0)	60.0 (45–70)	10–90
**55–59 years**	**Male (*n*** **= 86)**	52.0 (18.1)	45.0 (40–70)	10–90	53.5 (18.1)	50.0 (40–70)	0–90
	**Female (*n*** **= 86)**	54.6 (17.6)	50.0 (40–70)	10–90	56.5 (16.9)	51.2 (45–70)	10–90
**60–64 years**	**Male (*n*** **= 58)**	49.7 (18.6)	50.0 (40–65)	10–90	52.0 (18.1)	50.0 (40–65)	10–90
	**Female (*n*** **= 55)**	53.2 (19.2)	55.0 (40–70)	10–90	54.6 (20.2)	55.0 (40–73)	10–90
**65–69 years**	**Male (*n*** **= 62)**	49.9 (16.0)	45.0 (40–60)	10–90	51.9 (18.1)	50.0 (40–65)	10–90
	**Female (*n*** **= 61)**	54.8 (18.1)	56.6 (45–70)	10–90	56.9 (18.0)	55.0 (45–72)	20–90
**70–74 years**	**Male (*n*** **= 49)**	49.5 (19.3)	50.0 (40–65)	0–85	50.4 (14.9)	50.0 (40–70)	20–85
	**Female (*n*** **= 54)**	51.1 (18.2)	50.0 (40–65)	5–90	51.0 (18.7)	53.5 (40–70)	10–90
**75–79 years**	**Male (*n*** **= 52)**	47.6 (15.4)	45.0 (37–60)	15–90	50.4 (14.9)	45.0 (40–60)	20–85
	**Female (*n*** **= 46)**	45.9 (18.2)	45.0 (32–56)	5–90	51.0 (18.7)	50.0 (40–65)	10–90
**80–84 years**	**Male (*n*** **= 31)**	50.3 (19.4)	50.0 (34–60)	10–90	53.0 (18.8)	50.0 (42–70)	5–90
	**Female (*n*** **= 51)**	50.9 (19.1)	45.0 (40–65)	5–90	53.8 (18.0)	50.0 (40–65)	10–90
**85 years and over**	**Male (*n*** **= 10)**	46.9 (17.7)	45.0 (30–64)	25–75	51.5 (19.5)	50.0 (46–66)	20–80
	**Female (*n*** **= 10)**	47.8 (26.6)	65.0 (20–68)	15–80	48.6 (26.8)	65.0 (20–75)	10–80

### The effect of hand dominance

Table 4 shows the mean and SD for active flexion, abduction and external rotation of the left and right shoulders by hand dominance. There was a statistically significant difference (*p* < 0.05) in the range of active left and right shoulder flexion and left shoulder external rotation for those who were right hand dominant compared to those who were left hand dominant (Table 4).

**Table 4:** Overall mean (SD) shoulder movement in degrees by hand dominance

	Right hand (***n*** = 2125)			Left hand (***n*** = 257)		
	Mean (SD)	Median (IQR)	Range	Mean (SD)	Median (IQR)	Range
**Left shoulder flexion**	158.1 (18.3)*	160.0 (150–170)	0–180	161.9 (17.4)*	166.0 (150–177)	60–180
**Right shoulder flexion**	159.8 (18.7)*	162.0 (150–172)	10–180	161.7 (21.0)*	166.0 (154–176)	0–180
**Left shoulder abduction**	148.4 (20.6)	152.0 (140–160)	0–180	151.1 (16.5)	150.0 (140–162)	70–180
**Right shoulder abduction**	150.6 (20.1)	154.0 (140–164)	0–180	151.1 (20.9)	156.0 (140–164)	0–180
**Left shoulder external rotation**	56.4 (18.0)*	55.0 (45–57)	0–90	58.2 (17.1)*	60.0 (45–75)	10–90
**Right shoulder external rotation**	58.8 (17.8)	60.0 (45–75)	0–90	58.4 (17.7)	60.0 (45–75)	0–90

*Statistically significant difference in active range between those who are right and left handed, Mann-Whitney U test, *p* < 0.05

In addition, using hand dominance, flexion, abduction and external rotation of the dominant and non-dominant sides were determined. The mean range of dominant side flexion was 159.8° (SD 19.0°) and non-dominant side flexion was 158.4° (SD 18.7°); for dominant side abduction 150.4° (SD 20.0°) and non-dominant side 148.6° (SD 20.4°); and external rotation was 58.6° (SD 17.7°) and 56.5° (SD 17.9°) for the dominant and non-dominant sides respectively. The difference between the two sides was statistically significant (*p* < 0.001).

### The effect of diabetes

Table 5 presents the data for the mean and SD for active flexion, abduction and external rotation of the left and right shoulder according to diabetes status. There was statistically significant higher mean range (*p* < 0.05) for all assessed movements among those without diabetes compared to those with diabetes after adjustment for both age and sex.

**Table 5:** Overall mean range of shoulder movement in degrees for those with and without diabetes adjusted for age and sex

	No diabetes (***n*** = 2229)	Diabetes (***n*** = 150)
	Mean (SD)	
**Left shoulder flexion**	158.9 (16.3)*	154.2 (16.7)*
**Right shoulder flexion**	160.3 (17.2)*	156.0 (17.7)*
**Left shoulder abduction**	149.1 (18.2)*	143.3 (18.7)*
**Right shoulder abduction**	151.0 (18.5)*	144.2 (19.1)*
**Left shoulder external rotation**	56.9 (17.0)*	52.5 (17.6)*
**Right shoulder external rotation**	59.0 (17.0)*	55.0 (17.5)*

*Statistically significantly different active range between those with and without diabetes, adjusted for age and sex, MANOVA *p* < 0.05

## Discussion

Our study is the first to provide representative data of shoulder ROM from a large population-based sample of both males and females. This study indicates that in the general population and across all age groups, mean active shoulder flexion and abduction is greater in males, while active external rotation is greater in females. Mean ROM in all planes is lower among people with diabetes, and declines with age. There is also a lower mean range for all assessed movements except external rotation of the right shoulder for those who are right hand dominant compared to those who are left hand dominant.

Additional file 1 puts this study into context with previous studies [9–14]. The impact of sex on ROM in other studies has been variable with McIntosh et al. [9] and Gill et al. [10] reporting no effect of sex on ROM when using a convenience samples of 41 and 72 participants respectively, and Barnes et al. [11] demonstrating a higher ROM for females for all movements with a sample size of 140 males and 140 females but with children included in the sample. McIntosh et al. [9] suggest that generally women have been shown to have greater ROM than men, however the current study suggests that this may not be the case. It may be that the smaller sample size and different age ranges of each of these studies limited the ability to detect gender differences and also that the movements examined may have a gender specific bias. ROM may depend on the activities of daily living that are undertaken by males and females. In addition, there also may be an impact as a result of differences in hormonal effects on muscle, ligament, cartilage and bone [25].

Age is also a factor that determines ROM (Additional file 1). The decline in ROM across age groups shown in this study is consistent with other studies, [9–11] and likely to be attributable to age-related changes in the musculoskeletal system such as reductions in cartilage resilience, reduced elasticity of the ligaments, a reduction in muscle strength and changes in fat distribution [25, 26]. Occupation is acknowledged as a risk factor for shoulder disorders due to exposures such as working overhead, repetitive tasks and heavy lifting [27]. The cumulative impact of these activities as people age may also potentially reduce ROM through the mechanisms described above (reductions in cartilage resilience and reduced ligament elasticity).

McIntosh et al. [9] and Gill et al. [10] both highlighted that the AAOS ROM data [6] are often used to provide baseline measures for shoulder range. However, as Table 6 indicates, a range of 180° for abduction and flexion and 90° are not generally achieved in the population. Previous work [18], although focussing on the ROM of those who reported that they had shoulder pain and/or stiffness, also compared this range to those who have never had shoulder pain/stiffness. On average, there was approximately a 10% loss of ROM due to the presence of previous shoulder symptoms, but overall mean flexion range, adjusted for age, sex, BMI and current smoking was 159.2° and 157.7° for the right and left shoulders respectively, 149.7° and 147.7° mean abduction range and 58.1° and 56.0° of external rotation for the right and left shoulders respectively. These values are similar to the mean values presented in Tables 1, 2 and 3 for flexion, abduction and external rotation respectively.

In line with other studies, the results obtained from examining range of movement according to hand dominance and comparing dominant and non-dominant sides showed some small differences in range that while statistically significant are not likely to be clinically significant [11, 12, 14]. While Günal et al. [14] suggested that lower range of movement in the dominant side may be due to degenerative or ligamentous changes that are greater in that side compared to the non-dominant side, the current study demonstrated higher average range of movement for the dominant side but lower average range of movement for right handed people compared to left handed people. However, the study by Günal et al. focussed on a sample of males from the military and it may be that there is a minimal difference between dominant and non-dominant sides in a general community sample.

Those with diabetes (both self-reported and/or a fasting plasma glucose level of > = 7.0 mmol/l) also demonstrated a lower ROM compared to those without diabetes. While shoulder pain has been shown to be associated with diabetes, those who had ever reported shoulder pain and/or stiffness on most days for at least month were excluded from this study. Thus the reduced shoulder range of those with diabetes supports previous work which suggests that there are changes in the extracelluar matrix of the connective tissue in those with diabetes [28–30] resulting in a reduction in the collagen content and deficiencies in cross-linking [29] leading to a greater risk of reduced ROM.

### Strengths and limitations

The strengths of this study are the large sample size, with over 2000 randomly selected subjects of all ages participating in the study, thus providing normative values for ages 20 to 91 years. Measurements using standardised protocols and equipment with demonstrated reliability were undertaken in a clinic setting using staff trained by an experienced anthropometrist [22–24]. The study also excluded those who had ever reported shoulder pain and/ or stiffness on most days for over a month (encompassing those with chronic shoulder injuries or chronic conditions such as osteoarthritis) and those self-reporting a history of doctor-diagnosed rheumatoid arthritis. Information was also collected relating to whether participants had diabetes and this could be taken into consideration during analysis. Limitations of the study were that participants were asked if they had ever had shoulder pain and/or stiffness on most days for at least a month and thus the potential for recall bias exists. Participants were also not asked about the presence of shoulder pain for less than a month, thus some participants may have experienced short-lived shoulder pain. In addition, participants were not asked if they had undergone surgery for shoulder or upper limb problems, which may also impact on range of movement. In terms of measurement, no formal inter- or intra-rater reliability testing of staff was undertaken and measurement error was not determined. As a result, changes in range associated with treatment or over time may need to be interpreted with caution. Staff were also not experienced using the Plurimeter V inclinometer. Measurement of external rotation was only undertaken visually to the nearest 5° and also may be impacted by measurement error. Finally, three shoulder movements (adduction, internal rotation and extension) were not measured, due to time constraints on the clinic testing, thus normative data for these movements are not available from this sample.

## Conclusion

In conclusion, this study provides normative data for active shoulder flexion, abduction and external rotation, taken from a representative population-based community sample. We found that the overall average range of flexion for the right and left shoulders was between 157.1° and 161.5°; abduction was 147.7° to 151.5°; and external rotation was 55.0° to 60.9° among a general population sample (males and female). Knowing what constitutes “normal” range of active movement may provide reassurance and an indication of improvement for those with shoulder pain. Age and diabetes do impact on range and need to be considered in the diagnosis and management of shoulder conditions.

## Supplementary information

**Additional file 1.** Summary of shoulder ROM studies.

## Data Availability

Dr. Tiffany Gill and Professor Catherine Hill are both Chief Investigators for the North West Adelaide Health Study and as such, have access to all data that have been collected. However, as the North West Adelaide Health Study is also an ongoing cohort study, the data are not freely available. Data access can be granted upon request to the study Chief Investigators. Information relating to the data that have been collected is available at https://health.adelaide.edu.au/medicine/research/north-west-adelaide-health-study

## Abbreviations

AAOS: American Academy of Orthopedic Surgeons
CATI: Computer assisted telephone interview
GP: General practitioner
IQR: Interquartile range
MANOVA: Multiple analysis of variance
NWAHS: North West Adelaide Health Study
ROM: Range of movement
SD: Standard deviation
