# Supplementary material for: Shoulder range of movement in the general population: age and gender stratified normative data using a community-based cohort
Source: BMC Musculoskelet Disord. 2020 Oct 12;21:676. doi: 10.1186/s12891-020-03665-9 (PMC7549223; doi:10.1186/s12891-020-03665-9)
Supplement: Supplementary file 1 — Additional file 1. Summary of shoulder ROM studies. [file 12891_2020_3665_MOESM1_ESM.docx]

**Table 6: Summary of shoulder ROM studies**

|  | **Country** | **Population** | **Age** | **Numbers** | **Inclusion/**  **Exclusion** | **Method** | **Other information** | **Mean active shoulder flexion** | **Mean active shoulder abduction** | **Mean active shoulder external rotation** |
| --- | --- | --- | --- | --- | --- | --- | --- | --- | --- | --- |
| Adelaide | Australia | Random population based longitudinal cohort study | 20 years and over | 2404 | Inclusion:  Ever had shoulder pain and/or stiffness on most days for more than a month  Self-reported, doctor diagnosed rheumatoid arthritis | Active shoulder flexion, abduction, external rotation in standing | Height, weight, hand dominance, wide range of demographic characteristics, occupation, physical activity | Male left 159.9°  Right 161.5°  Female left 157.1°  Right 158.5° | Male left 149.7°  Right 151.5°  Female left 147.7°  Right 149.7° | Male left 55.0°  Right 55.0°  Female left 58.5°  Right 56.8° |

**Table 6: Summary of shoulder ROM studies (cont)**

|  | **Country** | | **Population** | **Age** | **Numbers** | | **Inclusion/**  **Exclusion** | | **Method** | **Other information** | **Mean active shoulder flexion** | **Mean active shoulder abduction** | **Mean active shoulder external rotation** |
| --- | --- | --- | --- | --- | --- | --- | --- | --- | --- | --- | --- | --- | --- |
| McIntosh et al.^9^ | Australia | Convenience sample, community dwelling.  Snowball sampling, chain referral | | 50 years and over | | 41 (21 male, 20 female) | | Inclusion: Independent activities of daily living, able to do sit-to-lie  transfers, no previous/  current impairment or tissue damage to shoulder | Active and passive flexion (seated), abduction (seated), internal/ external rotation (supine), arm abducted to 90°, elbow 90° flexion) measured with universal goniometer | Work history, current level of activity, height, weight hand dominance | Averaged left and right 132.2° | Averaged left and right 147.5° | Averaged left and right 80.2° |

**Table 6: Summary of shoulder ROM studies (cont)**

|  | **Country** | **Population** | **Age** | **Numbers** | **Inclusion/**  **Exclusion** | **Method** | **Other information** | **Mean active shoulder flexion** | **Mean active shoulder abduction** | **Mean active shoulder external rotation** |
| --- | --- | --- | --- | --- | --- | --- | --- | --- | --- | --- |
| Gill et al.^10^ | Australia | Convenience sample, community dwelling.  Snowball sampling. | 20-49 years | 72 (35 male, 37 female) | Inclusion: Independent activities of daily living, able to do sit-to-lie  transfers, no previous/  current impairment or tissue damage to shoulder | Active and passive flexion (seated), abduction (seated), internal/ external rotation (supine, arm abducted to 90°, elbow 90° flexion) measured with universal goniometer | Current level of activity, height, weight | Averaged left and right 158.9° | Averaged left and right 163.9° | Averaged left and right 84.9° |

**Table 6: Summary of shoulder ROM studies (cont)**

|  | **Country** | **Population** | **Age** | **Numbers** | **Inclusion/**  **Exclusion** | **Method** | **Other information** | **Mean active shoulder flexion** | **Mean active shoulder abduction** | **Mean active shoulder external rotation** |
| --- | --- | --- | --- | --- | --- | --- | --- | --- | --- | --- |
| Barnes et al.^11^ | United States | Not stated | 4-70 years | 280 (20 male, 20 female in each 10 year age category from 0-70) | Exclusion: History of shoulder surgery, dislocation  or fracture, or recent shoulder pain | Active and passive forward elevation (supine), abduction (supine), internal/ external rotation in 90° abduction (supine), external rotation in adduction (supine), extension (prone) with standard goniometer | Race, occupation  type, sports participation, throwing arm, writing hand | Female dominant 176.7°,  non-dominant 176.2°  Male dominant 173.6°,  non-dominant 173.5° | Female dominant 187.6°,  non-dominant 188.6°  Male dominant 180.1°,  non-dominant 181.8° | Female dominant 81.4°,  non-dominant 77.2°  Male dominant 78.3°,  non-dominant 73.7° |

**Table 6: Summary of shoulder ROM studies (cont)**

|  | **Country** | **Population** | **Age** | | **Numbers** | | **Inclusion/**  **Exclusion** | | **Method** | | **Other information** | | **Mean active shoulder flexion** | **Mean active shoulder abduction** | **Mean active shoulder external rotation** |
| --- | --- | --- | --- | --- | --- | --- | --- | --- | --- | --- | --- | --- | --- | --- | --- |
| Machedo and Magee^12^ | Canada | University staff and students and surrounding population with advertisement | | 18-59 years | | 90 females | | Exclusion:  Neurologic,  systemic, peripheral or rheumatic conditions  History of musculoskeletal injury in the past year  Surgery  Physical therapy treatment in the last year  Pregnant or having been pregnant in the past year  Involved in a high or professional level of sports  Diseases that might affect the level of the body hormones | | Active and passive abduction, flexion, internal/ external rotation (all supine), extension (prone) with goniometer | | Dominant side, occupation, height, weight, other demographics (not stated) | Only mean differences between sides reported. | | |

**Table 6: Summary of shoulder ROM studies (cont)**

|  | **Country** | **Population** | **Age** | **Numbers** | **Inclusion/**  **Exclusion** | | **Method** | **Other information** | **Mean active shoulder flexion** | **Mean active shoulder abduction** | **Mean active shoulder external rotation** |
| --- | --- | --- | --- | --- | --- | --- | --- | --- | --- | --- | --- |
| Boone and Azen^13^ | United States | Clinic population | 1-54 years | 109 males, 56  over 19 years | Exclusion: History of musculoskeletal or neural lesions | Active horizontal flexion, extension, neutral abduction (supine), forward flexion, inward/ outward rotation (all supine), extension (prone) with ordinary goniometer | | Race, hand dominance | 165.0° | 182.7° | 99.6° |

**Table 6: Summary of shoulder ROM studies (cont)**

|  | **Country** | **Population** | **Age** | | **Numbers** | **Inclusion/**  **Exclusion** | | **Method** | **Other information** | **Mean active shoulder flexion** | **Mean active shoulder abduction** | **Mean active shoulder external rotation** |
| --- | --- | --- | --- | --- | --- | --- | --- | --- | --- | --- | --- | --- |
| Gunal et al.^14^ | Turkey | Assessment for military service | 18-22 years | 1000 males | | Exclusion: illness or injury involving any joint of either  upper extremity | Passive abduction/ active glenohumeral abduction, Active and passive adduction, inward/ outward rotation, horizontal flexion, horizontal inward rotation (supine), active extension (prone) with goniometer | | Dominance (all right hand dominant) | No elevation measured | Passive neutral abduction and gleno-humeral abduction measured separately | Mean right 65.9°  Left 69.6° |
